# Survival Outcomes in Metastatic Colorectal Cancer Patients with *BRAF* Mutations Undergoing Targeted Chemotherapy: A Reconstructed Individual Patient Data Meta-Analysis of Randomized Controlled Trials

**DOI:** 10.1007/s12029-026-01597-6

**Published:** 2026-10-30

**Authors:** Syeda Kanza Kazmi, Muhammad Abdul Rehman, Hanaa Tariq, Aqsa Kabir, Jalib Ahmed, Nelson Shu-Sang S. Yee

**Affiliations:** 1 Department of Internal Medicine, Penn State Health Milton S. Hershey Medical Center, Hershey, United States; 2 Mayo Clinic School of Graduate Medical Education, Mayo Clinic, Rochester, MN United States; 3 Division of Gastroenterology & Hepatology, Mayo Clinic, Rochester, MN United States; 4 Department of Internal Medicine, University of Central Florida, Tallahassee, FL USA; 5 Sindh Medical College, Jinnah Sindh Medical University, Karachi, Pakistan; 6 Dow Medical College, Dow University of Health Sciences, Karachi, Pakistan; 7 Division of Hematology & Oncology, Department of Internal Medicine, Penn State Health Milton S. Hershey Medical Center, Hershey, United States

**Keywords:** BRAF V600E mutation, Metastatic colorectal cancer, Targeted Therapy, Anti-EGFR, anti-VEGF, meta-analysis

## Abstract

**Background:**

BRAF V600E–mutant metastatic colorectal cancer (mCRC) represents an aggressive molecular subtype associated with poor prognosis and limited response to conventional chemotherapy. Although multiple targeted strategies, including anti-vascular endothelial growth factor (*VEGF*), anti-epidermal growth factor receptor (*EGFR*), and *BRAF*-directed therapies, are used in clinical practice, their comparative effectiveness remains uncertain. We performed a reconstructed individual patient–data (IPD) meta-analysis of randomized controlled trials (RCTs) to evaluate survival outcomes across targeted therapeutic classes in *BRAF*-mutant mCRC.

**Methods:**

A systematic search of PubMed, Embase, Scopus, and Cochrane databases was conducted through July 2025 to identify RCTs reporting survival outcomes for *BRAF*-mutant mCRC. Kaplan–Meier curves were digitized and IPD reconstructed using established methods. A one-stage meta-analysis using Cox proportional hazards models was performed to estimate pooled overall survival (OS) and progression-free survival (PFS). Models were adjusted for study-level covariates and clustering, with Bonferroni correction applied for multiple comparisons.

**Results:**

Eighteen RCTs comprising 11,931 participants were included. Pooled median OS was 22.3 months for *BRAF*-directed regimens, 17.1 months for *VEGF*-targeted therapy, and 10.1 months for EGFR-directed therapy across studies evaluating first-line therapy. Across first-line studies, *BRAF* inhibitor directed regimen was associated with significantly improved OS compared with *EGFR* inhibition (HR 0.43, 95% CI 0.31–0.60; *P* < 0.001) and improved PFS (HR 0.47, 95% CI 0.42–0.53; *P* < 0.001). *VEGF* inhibition also demonstrated superior OS compared with *EGFR* inhibition (HR 0.56, 95% CI 0.38–0.81; *p* < 0.001). *EGFR*-directed regimen consistently demonstrated the poorest survival outcomes across analyses.

**Discussion:**

In this reconstructed IPD meta-analysis, *BRAF*-directed therapies were associated with the most favourable survival outcomes, whereas *EGFR* directed regimen demonstrated limited clinical benefit in *BRAF*-mutant mCRC. These results highlight the importance of biologically aligned pathway-targeted therapy and provide comparative evidence to inform treatment selection in this aggressive molecular subtype.

**Supplementary Information:**

The online version contains supplementary material available at https://doi.org/10.1007/s12029-026-01597-6.

## Introduction

Colorectal cancer (CRC) is the third most common cancer worldwide and the second leading cause of cancer-mortality in the world [1]. The American Cancer Society projected an estimated 52,900 deaths due to CRC in 2025 [2]. Despite advancement in screening and treatment, metastatic colorectal cancer (mCRC) accounts for the majority of CRC-related deaths [3], with a 5-year survival rate of 16.2% [2].

mCRC is a heterogeneous malignancy characterised by various molecular subtypes. *BRAF*-mutated mCRC is seen in 8–10% of patients with mCRC [4]. Among these, *BRAF* V600E mutation, which constitutes > 90% of *BRAF*-mutant malignancies, represents a distinctly aggressive phenotype associated with poorer overall prognosis and worse response to standard chemotherapy [4, 5]. While historically mCRC treatment has largely been based on combination irinotecan or oxaliplatin-based chemotherapy regimens [6], over the past several years it has become increasingly clear that molecular-targeted therapies play a pivotal role in mCRC management. The National Comprehensive Cancer Network (NCCN) currently recommends testing for *BRAF* status, as well as *KRAS/NRAS* status, in mCRC patients in order to guide treatment [7].

The management of *BRAF* V600E-mutant mCRC remains a formidable challenge for oncologists, with a complex treatment landscape that has evolved rapidly over the past several years [8]. The anti-vascular endothelial growth factor (*VEGF*) agent bevacizumab, in combination with doublet or triplet chemotherapy, has historically been recommended as standard of care for first-line treatment of *BRAF*-mutant mCRC [7, 9]. This was supported by evidence from studies such as a phase II trial which demonstrated an increase in 6-month progression-free rate for *BRAF* V600E-mutant patients treated with FOLFOXIRI (folinic acid, fluorouracil, oxaliplatin, and irinotecan) combined with bevacizumab [10]. The prospective, randomized FIRE-4.5 trial also reinforced the superiority of bevacizumab-based chemotherapy compared to chemotherapy with the anti-epidermal growth factor receptor (*EGFR*) agent cetuximab for *BRAF* V600E-mutant mCRC [11].

Conversely, while anti-*EGFR* agents such as cetuximab or panitumumab are often used in combination with chemotherapy for *RAS*-wild type (wt) and *BRAF*-wt mCRC, their role in the treatment of *BRAF*-mutated mCRC has remained controversial [9]. Evidence has increasingly pointed towards *BRAF* mutation being a negative predictive marker for response to anti-*EGFR* therapy alone or in combination with chemotherapy [12, 13]. Similarly, *BRAF* inhibitors, when used alone, have not been shown to confer a significant benefit in the treatment of *BRAF*-mutant mCRC [14]. These findings highlight the complex nature of the MAPK signalling pathway and resistance mechanisms in this disease.

However, the treatment landscape shifted significantly after the results of the BEACON CRC study, which established the role of anti-*EGFR* therapy, along with the *BRAF* inhibitor encorafenib, as the standard of care for previously treated *BRAF*-mutant mCRC. The results of this trial demonstrated improvement in overall survival (OS), progression-free survival (PFS) and overall response rate (ORR), compared to standard chemotherapy regimens [15]. Building on these findings, the recent BREAKWATER trial showed significantly improved OS and PFS with encorafenib plus cetuximab and chemotherapy, compared to standard of care for previously untreated *BRAF* V600E-mutant mCRC as well [16]. Based on this evidence, new guidelines now recommend this regimen for first-line treatment of *BRAF*-mutant mCRC [17].

When taken together, these observations highlight that the management of *BRAF* V600E-mutant mCRC remains a moving target. While newer trials have enhanced treatment options, the choice of therapeutic strategy has become more complex, with the comparative effectiveness of these therapies remaining unclear. Furthermore, existing data largely relies on post-hoc subgroup analyses and small sample sizes, limiting the ability to draw meaningful comparative conclusions. Therefore, this reconstructed individual patient-data (IPD) meta-analysis was designed in order to enhance statistical power by pooling reconstructed individual patient data across randomized control trials (RCTs) and thereby increase sample size. In doing so, we aim to provide a more comprehensive analysis of survival outcomes in *BRAF*-mutant mCRC to further guide optimal evidence-based management.

## Methods

**Fig. 1:**
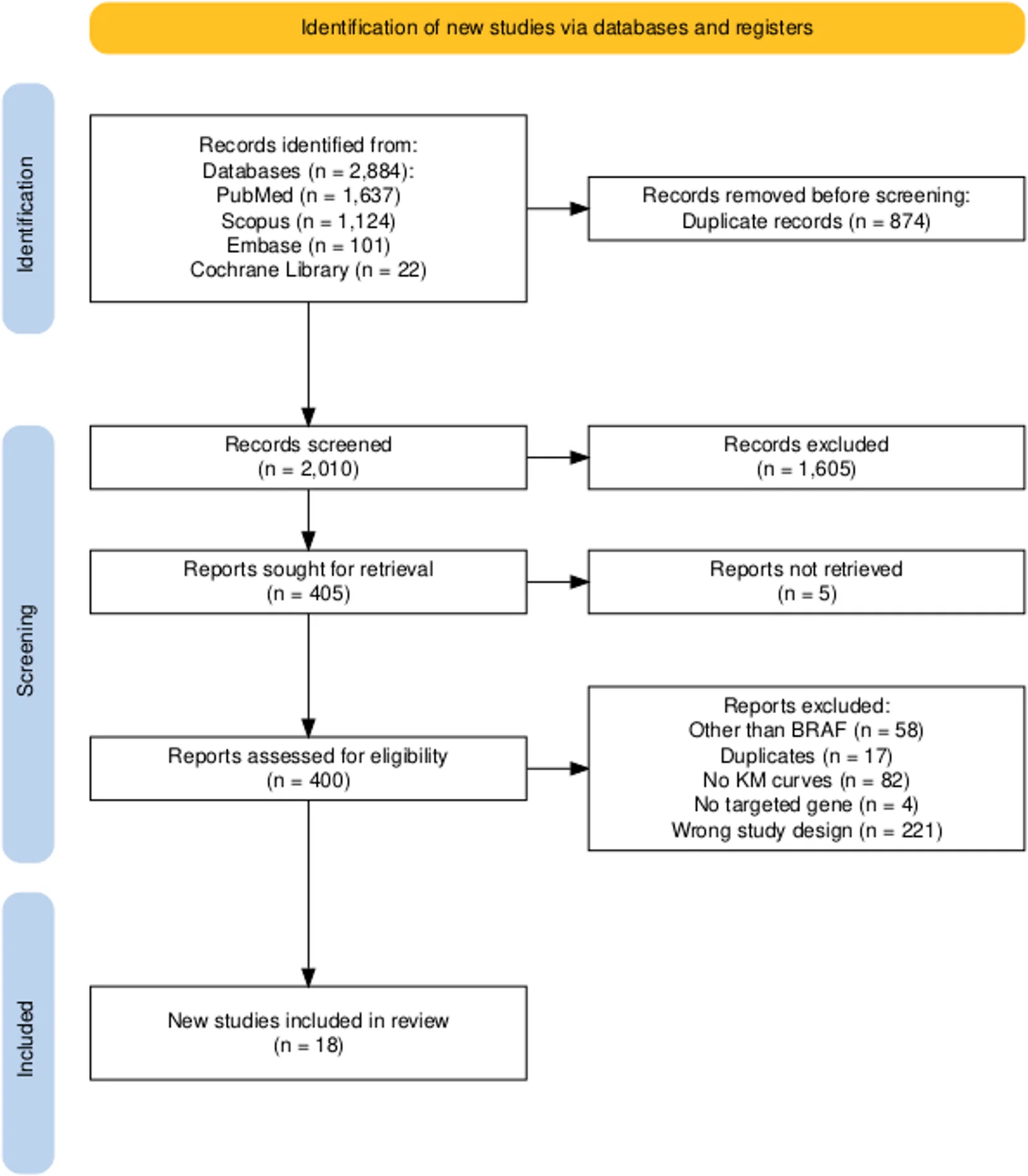
PRISMA flowchart

This review is reported as per the Preferred Reporting Items for Systematic Review and Meta-Analyses of individual participant data (PRISMA-IPD) reporting guidelines [18]. The PRISMA flowchart is shown in Fig. 1.

### Inclusion/Exclusion Criteria

To be eligible for this review, only RCTs evaluating targeted therapy for mCRC patients were considered. Trials had to report survival data for *BRAF*-mutated mCRC through at least one Kaplan-Meier curve and also provide the starting number at risk. Study designs other than RCTs, trials which did not evaluate targeted therapy, did not evaluate mCRC patients, or did not report at least one Kaplan-Meier curve were excluded.

### Search Strategy

In July 2025, we searched PubMed, Scopus, Embase, and Cochrane using major keywords “BRAF”, “metastatic colorectal cancer”, and “survival”. We did not limit our search to any particular year. Complete search strings for each individual database are present in the Supplementary File.

Articles found during the initial search were moved to Rayyan and then de-duplicated. Next, the articles were divided between two teams of two authors, each. Each team independently screened articles based on titles and abstracts. Short-listed articles were then reviewed by reading the full manuscript using a similar approach. At any stage, if the two authors disagreed on the eligibility of an article, a third author, outside the team, was consulted who made the final decision.

### Data Extraction

For eligible studies, we extracted data onto a pre-defined template. Data was independently extracted by AT, HT, and SKK. Any discrepancies were resolved independently by a third author.

Survival data for IPD was reconstructed using Kaplan-Meier curves in eligible studies. Kaplan-Meier curves were digitized via WebPlotDigitizer and redrawn [19]. Coordinates were converted to survival times and event indicators using established methods. Reconstructed curves were visually inspected and also verified numerically by comparing reconstructed median survival times against original values as shown in Supplementary Fig. 1.

Treatment arms were classified into therapeutic classes based on the primary targeted mechanism of action. ‘*BRAF*-directed regimens’ included any arm in which a *BRAF* inhibitor constituted the principal targeted agent, regardless of whether anti-*EGFR* therapy or chemotherapy was co-administered. ‘*EGFR*-directed regimens’ included arms using anti-*EGFR* agents without concurrent *BRAF* inhibition. ‘*VEGF*-directed regimens’ included arms using anti-*VEGF* agents as the primary targeted therapy.

### Risk of bias

We assessed risk of bias amongst included studies using the Cochrane risk-of-bias tool for randomized trials version 2 (RoB 2) [20].This tool evaluates five domains: randomization, deviations from intended interventions, missing outcome data, outcome measurement, and selection of reported results. Each domain is rated as either ‘low risk’, ‘some concerns’, or ‘high risk’. Risk of bias was independently performed by HT.

### Statistical Analysis

We conducted a one-stage meta-analysis to pool IPD data from eligible studies. Time-to-event was reconciled in months. We assessed proportionality by visually assessing Shoenfield’s residuals. If the assumption was not violated, we fit a proportional hazards Cox model. If the assumption was violated, we fit a time-varying Cox model. All models were adjusted for study-level covariables: sex, age, and study-level clustering. Microsatellite instability status, tumour-sidedness, line of therapy, ECOG performance status, RAS/co-mutation status, and post-progression treatment data were not consistently available across reconstructed IPD and could not be included as covariates (see Limitations).

We corrected for multiple comparisons using a Bonferroni adjustment and considered a *P*-value < 0.008 as significant. All analyses were conducted using R v4.5.2 and RStudio.

## Results

### Study Selection and Characteristics

Eighteen RCTs published between 2011 and 2025 met inclusion criteria, comprising predominantly multicentre phase II–III studies evaluating anti-*EGFR*-, anti-*VEGF*-, and *BRAF* inhibitor-containing strategies in mCRC [11, 13, 15, 16, 21–33]. Trials were conducted across Europe, North America, Australia, and multinational cooperative groups. Across studies, 11,931 patients were enrolled. Sample sizes ranged from 96 to 2,326 participants. The largest trial was CALGB/SWOG 80,405, followed by MRC COIN and the pooled analysis of CRYSTAL and OPUS. Molecularly selected *BRAF* V600E populations were evaluated primarily in BEACON CRC, SWOG S1406, and BREAKWATER. Across trials, the weighted median age approximated 62.4 years and women comprised approximately 39% of participants. The majority of patients (> 85%) had ECOG performance status 0–1. Colon primaries predominated. Among studies reporting metastatic distribution, liver metastases were present in approximately 63% of patients, followed by lung involvement; peritoneal metastases were less common. The summary of study characteristics is described in Table 1. Survival outcomes across therapy classes are shown in Table 2.

**Table 1:** Characteristics of included clinical trials evaluating targeted therapies in colorectal cancer

Study ID	Study Design	Trial	Country	Single Vs Multicenter	Sample Size	Intervention	Targeted Therapy Type	Known *BRAF* status	Known* BRAF*mutation
Maughan 2011	Randomized Phase 3	MRC COIN	UK and Ireland	Multicenter	1630	Arm A: Oxiplatin + Fluropyridimine Arm B: Oxiplatin + Fluoropyridimine + Cetuximab	Anti EGFR	1316	102
Price 2011	Randomized, Phase 3	AGITG MAX	Australia	Multicenter	471	Arm A: Capecitabine Arm B: Capectabine + Bevacizumab Arm C: Capecitabine+ Bevacizumab + Mitomycin	Anti VEGF	313	33
Modest 2012	Open label Randomized Phase II Trial	AIO KRK-0104	Germany	Multicenter	146	Arm A: Capecitabine + Irinotecan (CAPIRI) + Cetuximab Arm B: Capecitabine + Oxalipatin (CAPOX) + Cetuximab	Anti EGFR	146	17
Bokemeyer 2012	Pooled analysis of 2 RCTS	CRYSTAL + OPUS	Multinational	Multicenter	1535	Arm A : Chemotherapy (FOLFIRI/FOLFOX-4) Arm B: Chemotherapy (FOLFIRI/FOLFOX-4) + Cetuximab	Anti EGFR	800	70
Wasan 2014	Open-label, randomised, exploratory phase 2 trial	COIN-B	UK and Cyprus	Multicenter	226	Arm A: Intermittent FOLFOX + Intermittent Cetuximab Arm B: Intermittent FOLFOX + Continuous Cetuximab	Anti- EGFR	154	24
Cremolini 2015	Phase III, open-label, randomized	TRIBE	Italy	Multicenter	508	Arm A: FOLFOXIRI + Bevacizumab Arm B: FOLFIRI + Bevacizumab	Anti VEGF	193	28
Guren 2017	Phase III Clinical trial-Randomized	NORDIC-VII	Nordic Countries	Multicenter	566	Arm A: FLOX Arm B: FLOX + Cetuximab Arm C: Intermittent FLOX + continuous Cetuximab	Anti EGFR	457	55
Stintzing 2017	Phase III, open-label, randomized	FIRE-3 (AIO KRK-0306)	Germany	Multicenter	592	Arm A: FOLFIRI + Bevacizumab Arm B: FOLFIRI + Cetuximab	Anti VEGF, Anti EGFR	475	48
Modest 2019	Phase III, randomized, controlled, open-label	XELAVIRI	Germany	Multicenter	421	Arm A: Sequential escalation: Initial: Fluoropyrimidine (FP) + Bevacizumab; At first progression: CAPIRI + Bevacizumab or FOLFIRI + Bevacizumab Arm B: Upfront triplet: CAPIRI + Bevacizumab or FOLFIRI + Bevacizumab	Anti VEGF		22
Kurreck 2020	Randomized, open-label, phase II exploratory analysis of RCT	VOLFI trial (AIO KRK0109)	Germany	Single	96	Arm A: FOLFOXIRI + Bevacizumab Arm B: mFOLFOXIRI + Panitumumab	Anti EGFR	76	12
Kopetz 2021	Randomized, open-label, phase II clinical trial	SWOG S1406	USA	Multicenter	100	Arm A: Cetuximab + Irinotecan Arm B: Vemurafenib + Irinotecan + Cetuximab	Anti EGFR, BRAFinh	100	100
Moretto 2021	Randomized open label	TRIBE2	Italy	Multicenter	679 (66 for the study)	Arm A: FOLFOX + Bevacizumab, followed by FOLFIRI + Bevacizumab Arm B: FOLFOXIRI + Bevacizumab	Anti VEGF	646	66
Tabernero 2021	Phase III, randomized	BEACON CRC	Multinational	Multicenter	665	Arm A: Encorafenib + Binimetinib + Cetuximab Arm B: Encorafenib + Cetuximab Arm C: Cetuximab + Irinotecan OR Cetuximab + FOLFIRI	BRAFinh, MEKinh, Anti EGFR	665	665
Ten hoorn 2023	Randomized, Multicenter Phase III Trial	CAIRO2 Trial	Netherlands	Multicenter	736	Arm A: Capecitabine +Oxaliplatin + Bevacizumab + Cetuximab Arm B: Capecitabine + Oxaliplatin +Bevacizumab	Anti VEGF, Anti EGFR	522	249
Stintzing 2023	Randomized, open-label, phase II trial	FIRE-4.5	Germany	Multicenter	107	Arm A: mFOLFOXIRI + Cetuximab Arm B: mFOLFOXITI + Bevacizumab	Anti VEGF, Anti EGFR	107	107
Innocenti 2024	Randomized phase III clinical trial	CALGB/SWOG	USA	Multicenter	2326	Arm A: FOLFOX or FOLFIRI + Bevacizumab Arm B: FOLFOX or FOLFIRI + Cetuximab Arm C: FOLFOX or FOLFIRI + Cetuximab + Bevacizumab (discontinued early)	Anti EGFR, Anti VEGF	540	82
Tabernero 2024	Phase 3, randomized	AGENT Trial	Multinational	Multicenter	490	Arm A: Arfolitixorin + 5FU +Oxaliplatin + Bevacizumab Arm B: Leucovorin + 5FU + Oxaliplatin + Bevacizumab	Anti VEGF	240	31
Elez 2025	Open-label, randomized, phase 3 trial	BREAKWATER	Multinational	Multicenter	637	Arm A: Encorafenib + Cetuximab Arm B: Encorafenib + Cetuximab + mFOLFOX6 Arm C: mFOLFOX6 with or without bevacizumab, FOLFOXIRI with or without bevacizumab, or CAPOX with or without bevacizumab	Anti EGFR, Anti VEGF	637	600

Abbreviations: AIO, Arbeitsgemeinschaft Internistische Onkologie; AGITG, Australasian Gastrointestinal Trials Group; BRAF, B-Raf proto-oncogene; CAPIRI, capecitabine plus irinotecan; CAPOX, capecitabine plus oxaliplatin; CRC, colorectal cancer; EGFR, epidermal growth factor receptor; FOLFIRI, folinic acid, fluorouracil, and irinotecan; FOLFOX, folinic acid, fluorouracil, and oxaliplatin; FOLFOXIRI, folinic acid, fluorouracil, oxaliplatin, and irinotecan; FP, fluoropyrimidine; 5-FU, 5-fluorouracil; MEK, mitogen-activated protein kinase kinase; mFOLFOXIRI, modified FOLFOXIRI; mFOLFOX, modified FOLFOX; mFOLFOXITI, modified FOLFOXIRI; RCT, randomized controlled trial; UK, United Kingdom; USA, United States of America; VEGF, vascular endothelial growth factor. BEACON CRC, binimetinib, encorafenib, and cetuximab in BRAF-mutant colorectal cancer; CALGB, Cancer and Leukemia Group B; COIN, COntinuous chemotherapy plus cetuximab or INtermittent chemotherapy; FOLFIRI, folinic acid, fluorouracil, and irinotecan; FOLFOX-4, folinic acid, fluorouracil, and oxaliplatin regimen; MAX, capecitabine with or without bevacizumab and mitomycin; MRC, Medical Research Council; NORDIC-VII, Nordic colorectal cancer trial VII; OPUS, Oxaliplatin and Cetuximab in First-Line Treatment of mCRC; SWOG, Southwest Oncology Group; TRIBE, Triplet plus bevacizumab versus doublet plus bevacizumab in metastatic colorectal cancer; TRIBE2, Triplet plus bevacizumab followed by reintroduction versus sequential doublets plus bevacizumab; XELAVIRI, capecitabine and bevacizumab followed by irinotecan-based therapy versus upfront irinotecan-based therapy

**Table 2:** Survival outcomes in BRAF-mutant mCRC patients receiving chemotherapy subgrouped based on various monotherapy and combination therapy regimens. This analysis was limited to studies which evaluated first-line therapy. Survival models were adjusted for age, sex, and study-level clusters. HR: Hazard Ratio

	Studies, *N*	Patients, *n*	Total Events	Arm1 Events	Arm2 Events	HR (95% CI)	*P* value
Overall Survival							
*Monotherapy*							
BRAF vs. EGFR	8	746	462	201	261	0.43 (0.31–0.60)	< 0.001*
						Unclustered model^$^: 0.43 (0.33–0.57)	< 0.001*
VEGF vs. EGFR	12	648	517	256	261	0.56 (0.38–0.81)	< 0.001*
BRAF vs. VEGF	7	690	457	201	256	0.61 (0.31–1.20)	0.152
						Unclustered model^$^: 0.61 (0.42–0.90)	0.012
*Combination vs. monotherapy*							
BRAF vs. VEGF & EGFR	4	797	507	201	306	0.55 (0.52–0.58)	< 0.001*
BRAF vs. BRAF & EGFR	NA	NA	NA	NA	NA	NA	NA
EGFR vs. BRAF & EGFR	NA	NA	NA	NA	NA	NA	NA
Progression-Free Survival							
*Monotherapy*							
BRAF vs. EGFR	5	565	399	272	127	0.47 (0.42–0.53)	< 0.001*
						Unclustered model^$^: 0.47 (0.35–0.63)	< 0.001*
VEGF vs. EGFR	7	301	226	99	127	0.53 (0.42–0.68)	< 0.001*
BRAF vs. VEGF	5	524	371	272	99	0.68 (0.54–0.87)	<0.002*
						Unclustered model^$^: 0.68 (0.35–1.33)	0.265
*Combination vs. monotherapy*							
BRAF vs. VEGF & EGFR	3	767	567	272	295	0.73 (0.71–0.74)	< 0.001*
BRAF vs. BRAF & EGFR	NA	NA	NA	NA	NA	NA	NA
EGFR vs. BRAF & EGFR	NA	NA	NA	NA	NA	NA	NA

^$^Study-level clusters were not introduced because only 2025 Elez reported *BRAF*i monotherapy. Instead, an unclustered Cox model was used

*Model survived a Bonferroni correction

### Risk of Bias

**Fig. 2:**
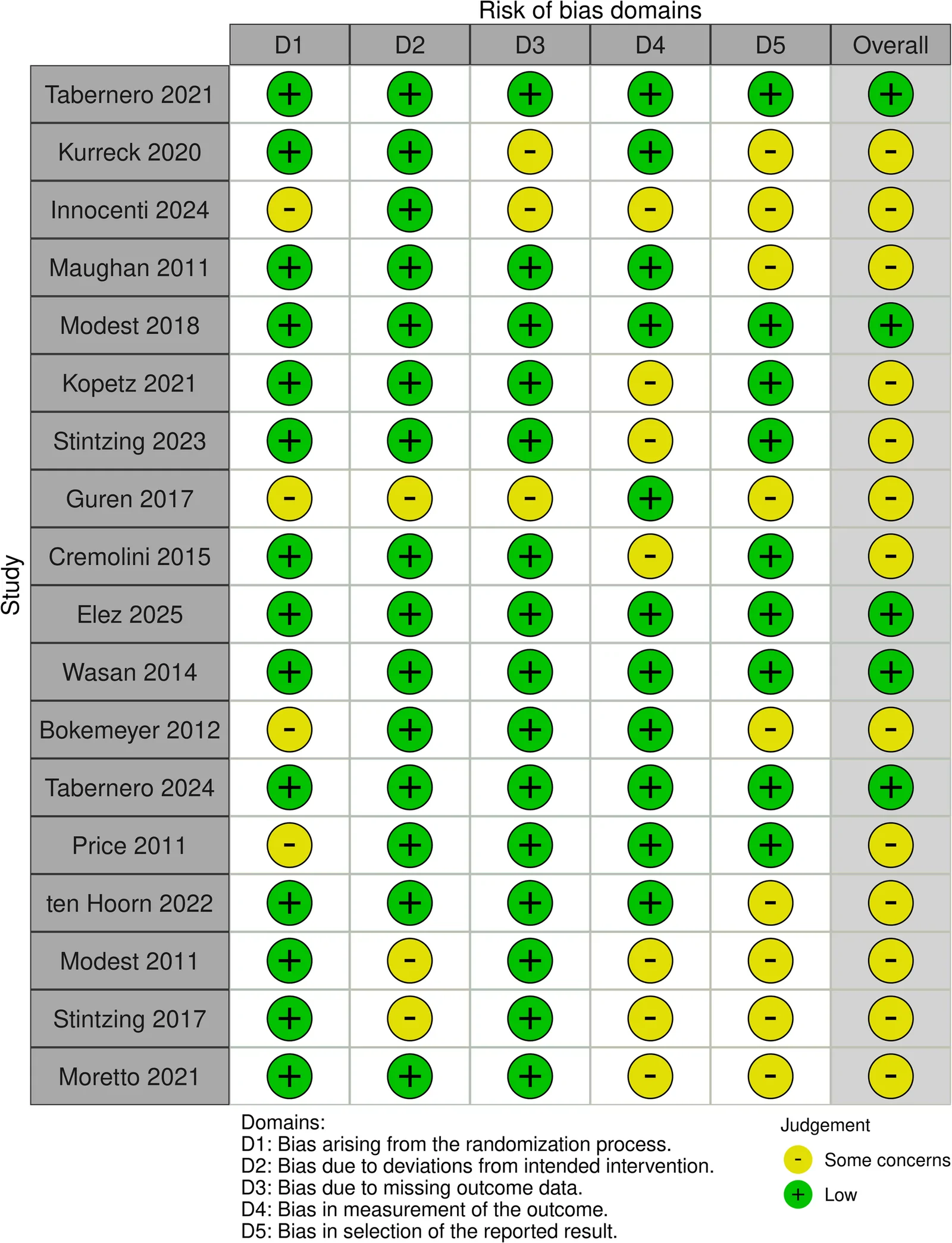
Traffic Light Plot of ROB

A summary of the risk of bias assessment is presented in Fig. 2. Overall, most studies were judged as having some methodological concerns, with only five studies achieving an overall low risk of bias. Domains four and five accounted for most concerns, primarily due to unblinded assessment of PFS, and uncertainty regarding whether BRAF-mutant-specific treatment comparison had been prespecified, respectively.

### Overall Survival

Pooled median OS across monotherapy therapeutic classes in studies evaluating first-line therapy were as follows: for *BRAF*-directed regimen it was 22.3 months (95% CI: 20.9–29.6). For *VEGF*-targeted regimens 17.1 months (95% CI 15.2–19.9) and for *EGFR*-targeted regimens 10.1 months (95% CI: 9.00-11.40).

### Monotherapy HR Comparison

**Fig. 3:**
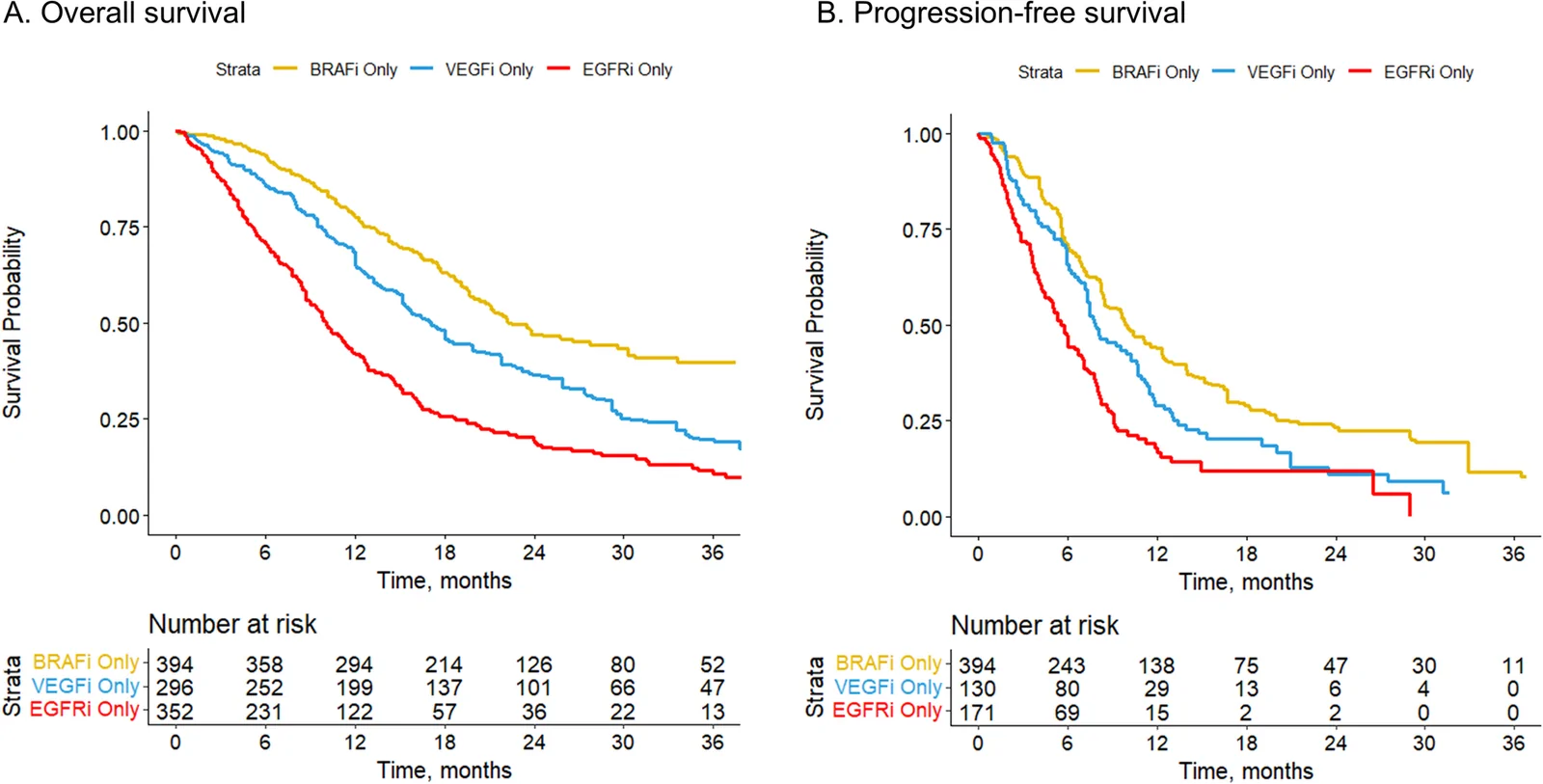
KM curves for mCRC patients who received monotherapy

Across studies evaluating first-line treatment, in 8 studies (*n* = 746 patients), *BRAF* inhibitor directed regimens were associated with statistically significant improvement in OS compared with EGFR directed regimens (HR 0.43, 95% CI 0.31–0.60; *P* < 0.001). Similarly, in 12 studies (*n* = 648), *VEGF* inhibitors demonstrated statistically significant improvement in OS compared with *EGFR* inhibitors (HR 0.56, 95% CI 0.38–0.81; *P* < 0.001). However, *BRAF* inhibitor directed regimen showed no statistically significant effect on OS versus *VEGF* inhibitor directed regiments (HR 0.61, 95% CI 0.31–1.20; *P* = 0.152) in a clustered model but suggest statistical significance in an unclustered model (*P* = 0.012). However, unlike other comparisons, the comparison of *BRAF*-inhibitors versus *VEGF*-inhibitors did not survive correction for multiple testing (Fig. 3).

### Combination Therapy Versus Monotherapy

Across studies evaluating first-line treatment, compared to dual *VEGF* and *EGFR* inhibition, therapy with *BRAF* directed regimen (4 studies; *n* = 797; 507 events) improved OS and the differences were statistically significant (HR 0.55, 95% CI 0.52–0.58; *P* < 0.001). This effect was also seen in the analysis which incorporated both first- and second-line studies. Although, after subgrouping to first-line studies there was no available data for other comparisons.

Across all studies, compared to *BRAF* and *EGFR* inhibition (3 studies; *n* = 887; 542 events), *BRAF* directed regimen suggested a benefit with statistically significant difference (HR 0.34, 95% CI 0.28–0.41; *P* < 0.001). In contrast, *EGFR* directed regimen versus *BRAF* plus *EGFR* therapy did not show a statistically significant difference in OS (9 studies; *n* = 1,116; 816 events; HR 1.25, 95% CI 0.94–1.68; *P* = 0.131).

Of note, this indirect comparison does not reflect head-to-head randomized evidence, and the apparent finding should not be interpreted as supporting *BRAF* directed regimen over established combination regimens. In addition, it is plausible that the analysis incorporating all studies is confounded by the presence of first- and second-line studies.

### Progression Free Survival

Regarding pooled median PFS, for *BRAF*-directed regimens, it was 9.9 months (95% CI 8.6–11.60), for *VEGF*-targeted regimens, 7.9 months (95% CI 7.3–10.6) and for *EGFR*-targeted regimens, 5.6 months (95% CI 4.8–7.1).

### Monotherapy HR Comparison

Across 7 studies, *BRAF* directed regimen was associated with a statistically significant improvement in PFS compared with *EGFR* directed regimen (HR 0.24, 95% CI 0.20–0.29; *P* < 0.001). This effect was also significant in the subgroup analysis limited to first-line studies (HR 0.47, 95% CI 0.42–0.53; *P* < 0.001). Similarly, *VEGF *directed regimen was associated with a statistically significant improvement in PFS compared with *EGFR *directed regimen (HR 0.46, 95% CI 0.33–0.64; *P* < 0.001) across all studies as well as in the subgroup analysis limited to first-line studies (HR 0.53, 95% CI 0.42–0.68; *P* < 0.001). There was no statistically significant difference in PFS between *BRAF* versus *VEGF *directed regimen (HR 0.68, 95% CI 0.35–1.33; *P* = 0.265) across all studies (Fig. 3).

### Combination Therapy Versus Monotherapy

Compared to dual *VEGF* and *EGFR* inhibition (3 studies; *n* = 767; 567 events), *BRAF*-directed regimen suggested a statistically significant improvement in PFS (HR 0.73, 95% CI 0.71–0.74; *P* < 0.001) across first-line studies. Likely confounded by the mix of first- and second-line studies, in the analysis involving both types of studies, *BRAF* directed regimen compared to combined *BRAF* and *EGFR* inhibition (3 studies) suggested a statistically significant PFS benefit (HR 0.43, 95% CI 0.41–0.47; *P* < 0.001). In contrast, *EGFR*-directed regimen versus *BRAF + EGFR* therapy was not associated with a statistically significant difference in PFS (HR 1.54, 95% CI 0.97–2.45; *P* = 0.068).

## Discussion

In this reconstructed IPD meta-analysis of eighteen RCTs, we provide a class-level survival comparison of targeted therapeutic strategies in *BRAF*-mutant mCRC, a biologically aggressive subtype with historically poor outcomes. Across pooled analyses, when compared against each other, a consistent survival gradient emerged: *BRAF*-directed regimens were associated with the most favourable OS and PFS, *VEGF*-targeted strategies demonstrated intermediate outcomes, and *EGFR* directed regimen was associated with the least favourable survival. Hazard-based comparisons confirmed statistically significant differences favouring *BRAF*-directed therapy over *EGFR* directed regimen for both endpoints and *VEGF*-directed therapy over *EGFR* inhibition for OS. These findings quantitatively define relative therapeutic performance across biologically distinct treatment classes in a molecularly enriched population.

The limited survival observed with *EGFR* directed regimen is biologically coherent. Constitutive activation of the MAPK pathway in *BRAF* V600E–mutated tumours renders upstream *EGFR* blockade insufficient to meaningfully suppress oncogenic signalling [34]. This intrinsic resistance mechanism has been consistently demonstrated in translational and clinical studies and is reflected in our pooled survival estimates [35]. Mechanistic studies have shown that *BRAF* inhibition in colorectal cancer paradoxically triggers rapid feedback reactivation of *EGFR*, which in turn reactivates *RAS* and *CRAF*-dependent MAPK signalling, a phenomenon far more pronounced in colorectal cancer than in *BRAF*-mutant melanoma, where *BRAF* inhibitor monotherapy is far more effective [36, 37]. This *EGFR*-driven reactivation has also been identified as a recurrent mechanism of acquired resistance in patients treated with RAF-inhibitor based regimens, with sequencing of post-progression tumours revealing *KRAS* and *BRAF* amplification and *MEK1* mutations that restore MAPK pathway flux [38]. Collectively, these findings explain why regimens combining *BRAF* and *EGFR* inhibition — which more durably suppress MAPK signalling than either agent alone — have translated into superior clinical outcomes, as demonstrated in BEACON CRC and BREAKWATER [39]. By contrast, therapies that directly target *BRAF*-driven signalling demonstrate closer alignment with tumour biology and correspondingly more favourable survival outcomes [40]. Although early phase studies of single-agent *BRAF* inhibitors in colorectal cancer showed modest durability of response, contemporary *BRAF*-directed strategies have evolved substantially, often incorporating rational pathway co-inhibition and optimized sequencing, which likely contributes to the improved survival distributions observed in modern datasets [14].

Importantly, the apparent superiority of *BRAF* directed regimen over *BRAF-EGFR* combination therapy in our pooled analyses across all studies should not be interpreted as evidence that monotherapy is clinically preferable. This finding is inconsistent with level 1 evidence from the BEACON CRC and BREAKWATER trials, both of which demonstrated superior efficacy with combined pathway inhibition in randomized head-to-head comparisons [15, 16]. The discordance in our analysis is a methodological artifact arising from the structure of indirect cross-trial comparisons: *BRAF* directed regimen data are derived predominantly from the BREAKWATER trial’s encorafenib–cetuximab doublet arm (which itself includes an anti*-EGFR* component and was paired with a strong chemotherapy comparator), while *BRAF–EGFR* combination data are drawn from trials with different patient populations, lines of therapy, and comparator-arm compositions. Hazard ratios estimated across trials are relative to their respective control arms and are therefore not directly comparable. This finding underscores a fundamental limitation of indirect treatment comparisons and should not be used to inform clinical decision-making. Indeed, after subgrouping to first-line studies, there was no available data to evaluate this relationship further.

*VEGF*-targeted therapy occupied an intermediate position in the survival gradient, with statistically significant differences in OS relative to *EGFR* inhibition but more modest and less consistent effects on PFS. This dissociation between radiographic progression and OS has been described in prior CRC studies and may reflect effects on tumour vascular normalization, metastatic dissemination, and facilitation of subsequent systemic therapy rather than direct cytotoxic impact [41–43]. However, much of the *VEGF* literature derives from molecularly unselected populations, and extrapolation to *BRAF*-mutant disease warrants caution [44]. Nonetheless, our findings support that angiogenesis inhibition continues to confer clinically meaningful survival benefits within this molecular subgroup.

Collectively, these results reinforce the central importance of biologically aligned therapy in *BRAF*-mutant mCRC. *EGFR* directed regimen demonstrates limited clinical utility in this context, whereas pathway-directed approaches yield more favourable outcomes. At the same time, our findings illustrate the complexity of synthesizing heterogeneous randomized data across evolving therapeutic eras. Differences in trial design, chemotherapy backbone, prior therapy exposure, and availability of post-progression treatment likely influence absolute survival estimates and may attenuate indirect class comparisons. These considerations are particularly relevant in *BRAF*-mutant disease, where prognosis is influenced by tumour sidedness, microsatellite instability, and co-mutation status; variables that could not be uniformly adjusted for in the present analysis [45–47].

This study has several notable strengths. Reconstructed IPD permitted consistent time-to-event modelling across trials and enabled direct hazard-based comparisons between therapeutic classes, improving precision relative to aggregate-level meta-analyses. Inclusion of exclusively RCTs enhances internal validity, and the large, pooled sample provides one of the most comprehensive contemporary survival syntheses in this molecularly defined population. However, limitations merit consideration. Reconstructed IPD cannot fully substitute for access to original patient-level datasets. Critically, our adjusted models accounted only for age, sex, and study-level clustering; several clinically important prognostic variables in *BRAF*-mutant mCRC including microsatellite instability status, tumour sidedness, line of therapy, ECOG performance status, RAS/co-mutation status, and post-progression treatment exposure, were unavailable at the reconstructed IPD level and could not be incorporated into the models. Because these variables are known to independently influence survival in this population and may be unevenly distributed across therapeutic classes and trials, their omission represents a substantial constraint on causal interpretation: the HRs reported here should be understood as adjusted class-level associations rather than causally identified treatment effects. We acknowledge that the survival gradient described in this analysis is derived from indirect, cross-trial comparisons and therefore cannot establish comparative superiority with the same inferential strength as randomized head-to-head evidence. Included trials differed substantially in line of therapy, chemotherapy backbone, treatment era, patient selection criteria, comparator-arm composition, and availability of post-progression therapies all of which may confound class-level hazard estimates. Given this incomplete confounder adjustment, residual confounding cannot be excluded as a contributor to the observed between-class differences. Accordingly, the observed survival gradient should be interpreted as hypothesis-generating and descriptive of relative therapeutic performance across trials, rather than as definitive evidence of causal superiority of one class over another.

An additional source of heterogeneity is that several older included trials (e.g., MRC COIN, NORDIC-VII, CRYSTAL/OPUS) enrolled molecularly unselected populations, with *BRAF*-mutant subgroups identified through post-hoc analyses. These subgroups were often small, and the trials were not powered to detect treatment effects within the *BRAF-*mutant subset. In contrast, BEACON CRC and BREAKWATER prospectively enrolled *BRAF* V600E–mutant patients. This difference in molecular enrichment strategy may influence the precision and generalizability of survival estimates across therapeutic classes.

This meta-analysis characterizes relative survival patterns across targeted therapeutic classes in *BRAF*-mutant mCRC. While *BRAF*-directed strategies were associated with the most favourable survival profiles in pooled analyses, these findings are derived from indirect cross-trial comparisons and should be interpreted as complementary to, rather than a substitute for, randomized head-to-head evidence.

Current level 1 evidence from the BEACON CRC and BREAKWATER trials supports encorafenib-based combination therapy (with cetuximab ± chemotherapy) as the standard of care for *BRAF* V600E–mutant mCRC. The indirect comparisons presented herein are exploratory and hypothesis-generating and do not supersede direct randomized evidence. Continued prospective, biomarker-stratified investigation will be essential to refine optimal sequencing and further improve outcomes in this high-risk population. 

## Supplementary Information

Below is the link to the electronic supplementary material.

Supplementary Material 1 (DOCX 526 KB)

## Data Availability

The datasets generated during and/or analysed during the current study are available from the corresponding author on reasonable request.
